# 单中心成人重型血友病A低/中剂量凝血因子Ⅷ预防治疗30例疗效分析

**DOI:** 10.3760/cma.j.issn.0253-2727.2023.01.007

**Published:** 2023-01

**Authors:** 燕慧 袁, 佩佩 许, 岳一 徐, 莎 刘, 晓雁 邵, 玮婧 张, 黎 龚, 敏 周, 兵 陈, 荣富 周

**Affiliations:** 1 南京大学医学院附属南京鼓楼医院血液科，南京 210008 Department of Hematology, the Affiliated Drum Tower Hospital of Nanjing University Medical School, Nanjing 210008, China.; 2 南京大学医学院附属南京鼓楼医院超声科，南京 210008 Department of Ultrasound, the Affiliated Drum Tower Hospital of Nanjing University Medical School, Nanjing 210008, China

**Keywords:** 血友病A, 预防治疗, 凝血因子Ⅷ, Hemophilia A, Prophylaxis, Coagulation factor Ⅷ

## Abstract

**目的:**

评估低/中剂量凝血因子Ⅷ（FⅧ）预防治疗在成人重型血友病A患者中的疗效。

**方法:**

纳入在南京大学医学院附属南京鼓楼医院接受低/中剂量FⅧ预防治疗的30例成人重型血友病A患者（低剂量预防组20例、中剂量预防组10例），对预防治疗前、后的年出血次数（ABR）、年关节出血次数（AJBR）、靶关节数量、血友病功能独立性评分（FISH）、生活质量评分及健康状况评分（SF-36）进行回顾性分析、比较。

**结果:**

中位随访48个月。与预防前按需治疗时相比，低、中剂量预防治疗组ABR、AJBR、靶关节数量均明显降低（*P*<0.05），且中剂量预防治疗组改善优于低剂量预防治疗组（*P*<0.05）。低、中剂量预防治疗后与按需治疗时相比，患者的FISH评分、生活质量评分及SF-36评分均明显改善（*P*>0.05），但低剂量预防组和中剂量预防组之间以上指标差异无统计学意义（*P*>0.05）。

**结论:**

低、中剂量FⅧ预防治疗较按需治疗可明显降低重型血友病A患者出血频率，延缓关节病变的进展，提高患者生活质量。中剂量预防组临床出血改善方面优于低剂量预防组。

## Abstract (en)

**Objective:**

To evaluate the clinical effects of low- and intermediate-dose factor Ⅷ（FⅧ）prophylaxis in Chinese adult patients with severe hemophilia A.

**Methods:**

Thirty adult patients with severe hemophilia A who received low-（*n*＝20）/intermediate-dose（*n*＝10）FⅧ prophylaxis at Nanjing Drum Tower Hospital affiliated with Nanjing University Medical College were included in the study. The annual bleeding rate（ABR）, annual joint bleeding rate（AJBR）, number of target joints, functional independence score of hemophilia（FISH）, quality of life score, and health status score（SF-36）before and after preventive treatment were retrospectively analyzed and compared.

**Results:**

The median follow-up was 48 months. Compared with on-demand treatment, low- and intermediate-dose prophylaxis significantly reduced ABR, AJBR, and the number of target joints（*P*<0.05）; the improvement in the intermediate-dose prophylaxis group was better than that in the low-dose prophylaxis group（*P*<0.05）. Compared with on-demand treatment, the FISH score, quality of life score, and SF-36 score significantly improved in both groups（*P*<0.05）, but there was no significant difference between the two groups（*P*>0.05）.

**Conclusion:**

In Chinese adults with severe hemophilia A, low- and intermediate-dose prophylaxis can significantly reduce bleeding frequency, delay the progression of joint lesions, and improve the quality of life of patients as compared with on-demand treatment. The improvement in clinical bleeding was better with intermediate-dose prophylaxis than low-dose prophylaxis.

血友病A是一种X染色体连锁的隐性遗传性出血性疾病[1]，主要表现为凝血因子Ⅷ（FⅧ）缺乏导致的反复发生的自发性关节及肌肉出血。目前血友病A的治疗主要是FⅧ替代治疗，分为按需治疗和预防治疗两种模式。按照预防治疗FⅧ的剂量和频率不同，可分为大剂量（25～40 U/kg每周3次）、中剂量（15～25 U/kg每周3次）及低剂量（10～15 U/kg每周2～3次）预防治疗方案[2]–[3]。对于存在关节损害的成人重型血友病A，国内长期预防治疗的临床数据较少。本研究通过对南京市30例重型血友病A患者进行低、中剂量预防治疗与按需治疗的数据对比，评估低/中剂量预防治疗在中国成人重型血友病A患者中的疗效。

## 病例与方法

一、病例来源

将2010年1月至2020年12月在南京大学医学院附属南京鼓楼医院接受低、中剂量FⅧ预防治疗的30例成年重型血友病A患者纳入本研究，患者预防治疗前均在本中心接受按需治疗至少1年。其中4例患者2010年开始预防治疗，其余26例2015年后开始预防治疗。对这些患者低、中剂量预防治疗前、后的临床资料进行回顾性分析、比较。所有患者均为男性，暴露日均>150 d，抑制物检测为阴性。患者在鼓楼医院血友病诊疗中心中位规律随访，治疗资料完整并知情同意。

二、研究方法

由血友病专科医师对患者填写的纸质或电子问卷进行分析并完成以下数据的收集、记录和整理：年出血率（ABR）、年关节出血率（AJBR）、靶关节、血友病功能独立性评分（FISH）、血友病Haem-QoL-A中文版评分及健康状况调查问卷（SF-36中文版），研究成人重型血友病A患者在低、中剂量预防治疗和按需治疗两种不同治疗方案下的出血情况、关节功能、生活质量变化等疗效指标及其变化规律。

三、观察指标

1. ABR及AJBR[4]：回顾性收集并记录患者随访期间按需或低、中剂量预防治疗时的出血情况，并进行年化处理。

2. 靶关节[4]：根据世界血友病联盟（WFH）对靶关节的定义，某一关节在连续6月内出血次数≥3次定义为靶关节，连续12月内该关节出血≤2次定义为靶关节解除。

3. FISH评分[5]：评价项目包括A（自我照顾）、B（移动）、C（运动）共8项，根据患者是否需要他人帮助完成将评分依次划分为4个等级，分值分别为1、2、3、4分，总分32分，评分越高，表明患者日常生活能力越好。

4. 血友病Haem-QoL-A中文版评分[6]：从日常活动、情绪和感受、工作学习以及治疗四个方面评估疾病对患者生活的影响。各条目采用5分计，“从不”计0分，“一直都会”计5分，其中9个条目需要反向计分。分数越高代表生活质量越差。

5. 健康状况调查问卷（SF-36中文版）[7]：本研究采用SF-36量表进行患者生活质量自评。SF-36共有9个维度和36个条目。先计算原始分数，再用标准公式计算转换分数。分数越高代表相应健康状况越好。

四、统计学处理

使用IBM SPSS 20.0统计软件对数据进行Kolmogorov-Smirnov正态性检验，符合正态分布、方差齐同的数据使用*t*检验进行比较，不符合正态分布的数据则使用Wilcoxon及Mann-Whitney秩和检验进行比较，*P*<0.05表示差异有统计学意义。

## 结果

一、患者临床情况

4例2010年开始预防治疗的患者为儿童患者，预防治疗持续至成年，2010年开始预防时年龄分别为9、10、10、15岁，2015年分别为14、15、15、20岁。其余26例2015年以后预防治疗的患者均为成年患者。截至2015年，接受低、中剂量预防治疗30例重型血友病A中位年龄30.5（14～52）岁；体重（63.8±11.2）kg，中位体重64（45～88）kg，中位BMI 22.4（16.1～29.1）kg/m^2^。历经数年预防治疗，2020年12月中位体重66（45～90）kg，中位BMI 23.3（16.1～30.8）kg/m^2^。中位FⅧ∶C基线活性为0.45％（0.1％～0.9％）。中位预防治疗时间48（6～126）月，11例患者预防治疗5年以上。

入组前6例患者既往因输血感染肝炎并存，其中5例丙型肝炎，1例乙型肝炎和丙型肝炎并存；5例患者曾发生颅内出血，其中2例遗留单侧肢体活动受限。

30例患者中有8例在本院行关节手术治疗（3例患者按需治疗时手术，5例患者预防治疗后手术）。其中髋关节置换术4例，髋关节以下截肢术1例，膝关节置换术2例，膝关节融合术1例。

二、按需治疗及预防治疗剂量

按需治疗很多患者是非及时、非足量的治疗，关节出血时给予血源性或重组FⅧ制剂30～40 U/kg，疗程1～3天。20例患者采用低剂量预防治疗方案，其中1例患者2010年即开始低剂量预防，19例2015年后开始低剂量预防，给予血源性或重组FⅧ制剂10～15 U/kg每周2～3次。10例患者采用中剂量预防治疗方案，其中3例患者2010年开始，7例2015年后开始，给予血源性或重组FⅧ制剂15～25 U/kg每周3次。

三、按需治疗与低、中剂量预防治疗时的临床出血情况

低剂量组和中剂量组预防治疗前（按需治疗阶段）ABR及AJBR差异无统计学意义（*P*>0.05）。预防治疗后低、中剂量组临床出血情况（ABR、AJBR、靶关节数量）较预防治疗前（按需治疗阶段）均明显改善（*P*<0.05），且中剂量预防治疗组较低剂量治疗组出血次数更少（*P*<0.05）。详见表1、表2。

**表1:** 重型血友病A患者低剂量凝血因子Ⅷ预防治疗前后临床出血情况比较［*M*（范围）］

组别	例数	ABR（次）	AJBR（次）	靶关节（个）
预防治疗前	20	45（8～68）	30（5～60）	4（1～6）
预防治疗后	20	15（2～35）	10（2～30）	2（0～4）

*z*值		−3.921	−3.921	−2.962
*P*值		0	0	0.003

注 ABR：年出血率；AJBR：年关节出血率

**表2:** 重型血友病A患者中剂量凝血因子Ⅷ预防治疗前后临床出血情况比较［*M*（范围）］

组别	例数	ABR（次）	AJBR（次）	靶关节（个）
预防治疗前	10	33（17～45）	25（12～40）	2.5（1～4）
预防治疗后	10	5（1～13）	3（1～10）	0（0～2）

*z*值		−2.805	−2.865	−2.701
*P*值		0	0.005	0.007

注 ABR：年出血率；AJBR：年关节出血率

四、FISH评分、Haem-QoL-A评分及SF-36评分

患者低、中剂量预防后较预防前FISH评分、Haem-QoL-A评分及SF-36评分均明显改善（*P*<0.05）。低剂量预防组和中剂量预防组相比FISH评分、Haem-QoL-A评分及SF-36评分差异无统计学意义（*P*>0.05），详见表3～5。

**表3:** 重型血友病A患者凝血因子Ⅷ预防治疗前后FISH评分（分，*x±s*）

组别	例数	预防治疗前	预防治疗后	*t*值	*P*值
中剂量预防组	10	19.6±3.6	25.5±3.8	3.783	0.001
低剂量预防组	20	19.0±3.8	23.7±4.1	3.771	0.001

**表4:** 重型血友病A患者凝血因子Ⅷ预防治疗前后Haem-QoL-A评分［分，*M*（范围）］

组别	例数	预防治疗前	预防治疗后	*z*值	*P*值
中剂量预防组	10	147（74～177）	64（15～101）	−2.803	<0.001
低剂量预防组	20	150（107～176）	72（25～101）	−3.921	<0.001

**表5:** 重型血友病A患者凝血因子Ⅷ预防治疗前后SF-36评分（分，*x±s*）

组别	例数	预防治疗前	预防治疗后	*t*值	*P*值
中剂量预防组	10	255.7±73.4	632.0±57.8	12.738	<0.001
低剂量预防组	20	222.8±38.9	615.5±59.3	24.772	<0.001

五、25例预防治疗期间未行手术治疗患者相关指标及生存质量评估比较

全部30例预防治疗患者中8例行关节手术，其中5例患者手术治疗发生于预防治疗期间。预防治疗期间未行手术治疗的25例重型血友病A患者出血相关指标及生存质量评分均优于预防治疗前（*P*值均<0.001）。详见表6。

**表6:** 25例预防治疗期间未行手术治疗重型血友病A患者出血相关指标及生存质量评估比较

指标	预防治疗前（25例）	预防治疗后（25例）	统计量	*P*值
ABR［次，*M*（范围）］	40（8～65）	8（1～35）	*z* =−5.186	<0.001
AJBR［次，*M*（范围）］	30（5～60）	5（1～30）	*z* =−5.172	<0.001
靶关节［个，*M*（范围）］	3（1～5）	1（0～4）	*z* =−3.483	<0.001
FISH评分（分，*x±s*）	19.2±3.9	24.4±4.2	*t* =4.464	<0.001
Haem-QoL-A评分［分，*M*（范围）］	149（74～175）	68（15～101）	*z* =−5.880	<0.001
SF-36评分（分，*x±s*）	232.5±46.2	624.5±52.9	*t* =27.900	<0.001

注 ABR：年出血率；AJBR：年关节出血率；FISH：血友病功能独立性评分

六、4例儿童预防延续至成年患者评估

4例2010年开始预防治疗的患者为儿童患者，其中3例为中剂量预防治疗，1例为低剂量预防治疗，成年后预防治疗仍未中断。该4例患者预防治疗后AJBR均小于5次，除1例患者因牙龈炎反复出现牙龈出血，另3例患者ABR均小于5次。

## 讨论

重型血友病A主要表现为患者反复发生自发的关节、肌肉出血甚至内脏出血，如治疗不及时会遗留严重的关节畸形、肌肉萎缩、运动障碍，严重者可能致残甚至致死。目前血友病A的治疗是以FⅧ替代治疗为主的综合治疗，而预防治疗是儿童的标准替代治疗方法[3]。

对于关节已经发育成熟，或者已存在关节损伤的成人预防治疗曾经存在争议。WFH 2020年第三版血友病管理指南指出：按需治疗无法阻止长期关节损害，任何剂量的预防治疗都优于按需治疗，青少年和成人患者建议进行三级预防在治疗，减缓关节损伤[8]。近年成人血友病预防治疗越来越受到关注，国外多项大型研究均显示FⅧ预防治疗可降低成人血友病A患者关节出血次数、减少关节损伤[9]–[12]。

国内血友病预防治疗的应用经验主要来自于儿童重型血友病A[13]–[14]，成人报道较少，成人长期预防治疗的报道更少见。2015年连小赟等[15]报道22例成人重型血友病A患者，低剂量三级预防治疗可以明显减少出血次数，改善关节状况及日常生活活动能力，提高生活质量。2016年李志涛等[16]报道成人重型血友病A低剂量预防治疗（16例）较按需治疗明显减少出血症状，FISH评分及SF-36评分改善。2018年孙雪岩等[17]报道了13例成人重型血友病3个月足量预防治疗，结果显示短期足量预防治疗可以显著改善患者出血情况。2021年陈蓉等[18]报告中、低剂量预防治疗16例成人重型血友病A患者，可以明显减少ABR、AJBR，改善关节功能。2021年孙竞等[19]对国内外成人预防治疗的文献进行分析综述，强调儿童过渡至青年后坚持预防治疗的重要性。本组患者低/中剂量预防治疗后ABR、AJBR、靶关节明显减少，FISH评分、Haem-QoL-A中文版评分及健康状况调查问卷（SF-36中文版）评分均明显改善，与上述报道一致。剔除5例预防治疗后行关节手术患者后再次分析仍同前一致。值得注意的是成人预防治疗后FISH评分改善较儿童预防治疗更加显著[16],[20]–[21]，考虑与成人预防治疗前出血导致整体关节功能较儿童患者更差、预防后关节功能改善更明显有关。

本组病例中剂量预防组ABR及AJBR明显低于低剂量预防组，提示更高剂量的预防治疗更能减少患者出血频率。然而10例中剂量预防组中有1例AJBR大于10次，20例低剂量预防组中有6例AJBR小于5次，说明血友病患者临床出血情况不仅与预防治疗剂量有关，其他因素如关节损害状态、患者的体力活动强度等因素也对出血倾向起重要作用。因此对于重型成人血友病A患者，除了需要更大剂量的FⅧ制剂进行预防治疗外，也需要结合关节状态和活动强度等进行个体化预防治疗，同时部分患者还需要考虑理疗、康复、手术等辅助治疗的干预，才能达到最佳治疗效果。

综上所述，本研究通过较长期的数据表明预防治疗对于关节稳定或者关节损害已形成的成人而言也是获益明显的。预防治疗较按需治疗可明显降低成人重度血友病A患者出血频率、延缓关节病变的进展、提高患者的生活质量。
